# Endoscopic‐Assisted Posterior Atlantoaxial Fusion: Technical Innovation and Clinical Experience

**DOI:** 10.1111/os.70358

**Published:** 2026-06-10

**Authors:** Jichong Zhu, Chengqian Huang, Jiang Xue, Jiarui Chen, Yufan Xu, Dequan Liu, Xinli Zhan, Chong Liu

**Affiliations:** ^1^ The First Affiliated Hospital of Guangxi Medical University Nanning China; ^2^ The Affiliated Hospital of Youjiang Medical University for Nationalities Baise China; ^3^ Key Laboratory of Molecular Pathology in Tumors of Guangxi Higher Education Institutions Baise China

**Keywords:** atlantoaxial instability, C1–C2 fixation, endoscopic‐assisted fusion, minimally invasive spine surgery, technical innovation

## Abstract

**Background:**

Atlantoaxial instability or dislocation requires reliable posterior stabilization and fusion to prevent neurological deterioration. Although conventional Goel–Harms posterior fixation provides effective biomechanical stability, it usually requires extensive posterior cervical muscle dissection and may be associated with substantial blood loss, postoperative axial pain, and soft‐tissue morbidity. Despite the advantages of minimally invasive endoscopic techniques in spinal surgery, clinical evidence regarding their application in the management of AAI/D remains limited. In this study, we report a clinical series of endoscopic‐assisted atlantoaxial lateral mass fusion, provide a detailed description of the key surgical steps, and evaluate its technical feasibility, safety, and early clinical outcomes.

**Methods:**

Sixteen patients diagnosed with AAI/D underwent endoscopic‐assisted posterior atlantoaxial fusion. The procedure utilized a uniportal endoscopic system through a 2.5–3.0 cm incision, enabling direct visualization of the C1–C2 lateral mass joint, precise screw placement, and intra‐articular bone grafting under continuous irrigation. Radiographic parameters, neurological function (JOA), pain (VAS), and neck disability (NDI) scores were assessed pre‐ and postoperatively.

**Results:**

All surgeries were successfully completed without intraoperative neurovascular injury or hardware malposition. The mean operative time was 103.06 ± 9.55 min, and the mean blood loss was 40.06 ± 9.28 mL. The atlantodental interval (ADI) decreased from 5.14 ± 1.51 mm preoperatively to 3.37 ± 0.52 mm postoperatively (*p* < 0.001), and C1–C2 joint height increased from 2.98 ± 0.27 mm to 4.37 ± 0.49 mm (*p* < 0.001). At 3 months, bone fusion was achieved in 93.8% of patients. Clinical outcomes improved compared with preoperative values, including JOA score (8.44 ± 0.89 to 13.31 ± 1.20), VAS score (8.00 ± 0.73 to 2.25 ± 0.45), and NDI score (31.88 ± 1.93 to 12.88 ± 1.26).

**Conclusion:**

This study introduces a novel minimally invasive technique for managing atlantoaxial instability using uniportal endoscopic‐assisted posterior fusion. The combination of direct endoscopic visualization, accurate screw–rod fixation, and controlled intra‐articular bone grafting offers reliable joint stabilization with reduced tissue disruption. Clinical outcomes suggest this approach may facilitate rapid recovery and improved perioperative safety, offering a valuable addition to current surgical strategies for AAI/D.

## Graphical abstract

Endoscopic‐assisted posterior atlantoaxial lateral mass fusion is introduced as a novel minimally invasive technique for treating atlantoaxial instability. The approach enables direct visualization, precise fixation, and effective bone fusion while minimizing muscle dissection, blood loss, and postoperative pain, achieving rapid neurological and functional recovery with high early fusion rates.

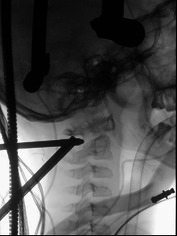

## 1 Introduction

Atlantoaxial instability or dislocation (AAI/D) represents a relatively common form of craniocervical junction instability that can result in compression of the upper cervical spinal cord and medulla oblongata [1]. If left untreated or improperly managed, this condition may lead to irreversible neurological deficits and even life‐threatening complications [2]. The distinctive anatomy of the atlantoaxial complex enables the vertebral bodies to avoid direct load bearing of the head; instead, mechanical forces are transmitted primarily through the lateral masses of C1 and C2. Consequently, the integrity and stability of the atlantoaxial lateral mass joints are crucial for maintaining normal craniovertebral biomechanics and physiological motion [3].

Over the past several decades, posterior atlantoaxial arthrodesis combined with screw‐rod fixation has become the gold standard for the surgical management of AAI/D [4, 5]. Nevertheless, the conventional Goel–Harms technique necessitates extensive dissection of the suboccipital and posterior cervical musculature, which is often associated with considerable intraoperative blood loss, postoperative axial pain, soft‐tissue injury, and impaired neck function [6]. These drawbacks highlight the need to develop novel, minimally invasive surgical techniques that can provide equivalent biomechanical stability while effectively reducing iatrogenic morbidity and promoting faster recovery [7, 8].

Recent advancements in imaging and navigation technologies have significantly promoted the widespread adoption of spinal endoscopic techniques, including full‐endoscopic surgery, uniportal split endoscopy, unilateral biportal endoscopy, and arthroscopic‐assisted uniportal spinal surgery [9]. These methods have been increasingly applied to decompression and fusion procedures of the lower cervical, thoracic, and lumbar spine [10]. Accumulating evidence indicates that minimally invasive endoscopic approaches achieve clinical outcomes comparable to those of conventional open surgery while offering notable advantages such as reduced complication rates, diminished tissue trauma, and faster postoperative recovery [11]. By minimizing soft‐tissue dissection and preserving anatomical structures, these techniques effectively reduce intraoperative blood loss and postoperative scar formation. Moreover, the direct proximity of the endoscope to the surgical target enables high‐definition visualization and precise intraoperative manipulation, facilitating meticulous hemostasis and accurate anatomical identification, thereby minimizing surgical trauma and optimizing clinical efficacy [12].

Despite the advantages of minimally invasive endoscopic techniques in spinal surgery, clinical evidence regarding their application in the management of AAI/D remains limited. In this study, we report a clinical series of endoscopic‐assisted atlantoaxial lateral mass fusion, provide a detailed description of the key surgical steps, and evaluate its technical feasibility, safety, and early clinical outcomes. Therefore, the objectives of this study were: (i) to describe the operative workflow and key technical details of endoscopic‐assisted posterior atlantoaxial lateral mass fusion; (ii) to evaluate the feasibility, safety, radiographic correction, fusion status, and early clinical outcomes of this technique in patients with atlantoaxial instability or dislocation; and (iii) to perform a preliminary comparison between this endoscopic‐assisted technique and conventional open posterior atlantoaxial fusion.

## 2 Materials and Methods

### 2.1 Patient Selection and Preoperative Evaluation

This study was approved by the Ethics Committee (2025‐E0882), and informed consent was obtained from all patients or their guardians. Sixteen patients diagnosed with AAI/D based on comprehensive radiological assessment and scheduled for posterior fusion were enrolled. Inclusion criteria were as follows: (i) radiologically confirmed atlantoaxial instability or dislocation requiring posterior C1–C2 fixation and fusion; (ii) complete preoperative radiographic evaluation, including X‐ray, CT/CTA, and MRI; (iii) availability of perioperative and follow‐up clinical data; and (iv) written informed consent provided by the patient or legal guardian. Exclusion criteria were as follows: (i) active spinal infection or tumor; (ii) severe congenital craniocervical deformity requiring complex reconstructive surgery; (iii) previous atlantoaxial surgery; (iv) incomplete clinical or radiographic data; and (v) inability to complete postoperative follow‐up. To provide a preliminary comparative reference, 20 patients who underwent conventional open posterior atlantoaxial fusion at the same institution were retrospectively included as a control group. These patients met the same diagnostic criteria and surgical indications as the endoscopic‐assisted group. The same exclusion criteria were applied to the open‐surgery cohort. Demographic characteristics, radiographic parameters, perioperative outcomes, and clinical scores were collected from the medical records and compared with those of the endoscopic‐assisted group. Because this comparison was retrospective and non‐randomized, the results were interpreted as exploratory.

Patient demographics, including sex, age, and diagnosis type, are summarized in Table 1. For transparency, our earlier technical case report has been cited because some illustrative intraoperative images from that previously published case were reused in Figure 3; however, that case was not included in the present study cohort or in the corresponding statistical analyses [13].

**TABLE 1:** Patient demographics and baseline characteristics.

No	Age (year)	Gender (M/F)	Height (cm)	Weight (kg)	Pre‐op ADI (mm)	Post‐op ADI (mm)	Pre‐op C1‐C2 joint height (mm)	Post‐op C1‐C2 joint height (mm)	Odontoid fracture	Transverse ligament injury	Disease duration (mo)
1	6	M	118	22	6.2	3.8	3.2	4.8	No	Yes	3
2	8	F	125	26	5.8	4.2	3.5	5.2	No	Yes	6
3	10	F	135	30	4.5	3.5	3	4.5	Yes	Yes	12
4	12	M	145	35	6.5	3.5	2.6	3.8	No	No	4
5	13	F	135	29	9.5	4.45	2.9	4	Yes	Yes	18
6	30	M	172	65	4	3	3.1	4.5	No	Yes	8
7	32	F	170	63	3.8	2.9	2.7	3.9	No	No	5
8	35	F	175	70	5.2	3.2	3.3	5	Yes	Yes	12
9	45	M	172	68	3.2	2.5	2.5	3.6	No	No	6
10	50	F	170	72	4.5	3	3	4.2	Yes	Yes	10
11	55	M	168	70	5	3.5	3.2	4.8	No	Yes	12
12	51	F	156	54	4.8	3.2	3	5	Yes	Yes	24
13	55	M	170	69	3.6	2.8	3	4.1	No	Yes	15
14	45	F	173	67	5.5	3.5	2.6	3.8	No	No	6
15	55	M	169	71	4.2	3	3.1	4.5	Yes	Yes	10
16	16	F	160	50	6	3.8	3	4.2	No	No	4

No	History of trauma	Congenital anomaly	Sensory disturbance	Motor dysfunction	JOA (Pre/Post/1 M/3 M)	VAS (Pre/Post/1 M/3 M)	Bone fusion at 3 M	NDI (Pre/Post 1 M/3 M)	Post‐op complications	Blood loss (mL)	Operation time (min)
1	No	No	Yes	Yes	8/13/14/15	8/4/3/2	Fused	32/20/15/12	None	25	95
2	No	No	Yes	Yes	7/12/13/11	7/3/3/2	Fused	30/18/14/10	None	60	100
3	Yes	No	Yes	Yes	9/14/15/13	9/5/4/3	Fused	28/19/15/12	Mild pain	35	110
4	No	No	Yes	Yes	8/13/14/13	8/4/3/2	Fused	33/21/16/13	None	50	90
5	Yes	No	Yes	Yes	10/15/16/15	8/4/3/2	Fused	35/22/17/14	None	40	125
6	No	No	Yes	Yes	9/14/15/14	9/5/4/3	Fused	31/20/15/13	Mild pain	45	105
7	No	No	Yes	Yes	8/13/14/12	7/4/3/2	Fused	30/19/14/12	None	35	98
8	Yes	No	Yes	Yes	10/15/16/15	8/4/3/2	Fused	34/21/16/14	None	50	120
9	No	No	Yes	Yes	9/14/15/14	7/3/3/2	Fused	32/20/15/13	None	35	102
10	Yes	No	Yes	Yes	8/13/14/13	8/4/3/2	Fused	33/21/16/14	None	40	108
11	No	No	Yes	Yes	9/14/15/14	9/5/4/3	Partial	31/20/15/13	Mild pain	30	100
12	Yes	No	Yes	Yes	7/12/13/12	8/4/3/2	Fused	35/22/17/15	Mild hematoma	45	105
13	No	No	Yes	Yes	8/13/14/13	7/4/3/2	Fused	32/20/15/13	None	30	97
14	No	No	Yes	Yes	9/14/15/14	8/4/3/2	Fused	31/19/15/13	None	35	92
15	Yes	No	Yes	Yes	8/13/14/13	9/5/4/3	Fused	33/21/16/14	Mild pain	36	107
16	No	No	Yes	Yes	8/13/14/12	8/4/3/2	Fused	30/18/14/11	None	50	95

*Note:* “Transverse ligament injury” refers specifically to acute ligamentous injury with definite signal alteration on preoperative MRI; chronic ligamentous laxity/insufficiency and non‐traumatic instability mechanisms were not coded under this variable.

**FIGURE 1:**
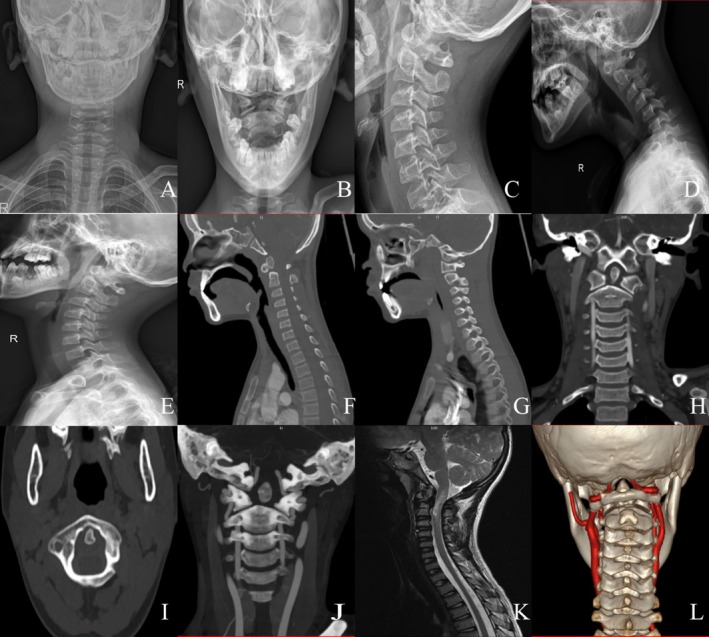
Preoperative radiographic, CT, CTA, and MRI evaluation of atlantoaxial instability/dislocation. (A) Anteroposterior cervical radiograph showing overall cervical alignment. (B) Open‐mouth odontoid radiograph showing the odontoid process and bilateral C1–C2 lateral mass joints. (C) Neutral lateral cervical radiograph showing the atlantodental interval and sagittal alignment. (D, E) Dynamic flexion–extension lateral radiographs used to evaluate atlantoaxial instability and reducibility. (F, G) Sagittal CT reconstruction showing the C1–C2 relationship and atlantoaxial alignment. (H) Coronal CT reconstruction showing the odontoid process and bilateral atlantoaxial joint alignment. (I) Axial CT image showing the odontoid process and C1 ring morphology. (J) Coronal CTA image showing the course of the vertebral arteries and their relationship with the osseous structures of the craniovertebral junction. (K) Sagittal MRI showing the spinal cord and craniovertebral junction, used to assess neural compression and associated abnormalities. (L) Three‐dimensional CTA reconstruction showing the posterior osseous anatomy and bilateral vertebral arteries. CT, computed tomography; CTA, computed tomography angiography; MRI, magnetic resonance imaging.

Cervical X‐rays were obtained in multiple views, including anteroposterior, lateral, open‐mouth odontoid, and dynamic flexion–extension positions. The anteroposterior view was used to assess overall cervical alignment and detect potential lateral displacement (Figure 1A). The lateral view allowed evaluation of the atlantodental interval (ADI), the spatial relationship between C1 and C2, and the sagittal alignment of the cervical spine (Figure 1C). The open‐mouth odontoid view provided direct visualization of the odontoid process and bilateral C1–C2 lateral mass joints, facilitating assessment of joint congruency and asymmetry (Figure 1B). The flexion–extension views were used to evaluate dynamic stability and the reducibility of atlantoaxial dislocation, providing essential information for preoperative planning and intraoperative positioning (Figure 1D,E).

Further imaging examinations (Figure 1F–L) included computed tomography (CT) with three‐dimensional reconstruction, CT angiography (CTA), and magnetic resonance imaging (MRI). Radiographic parameters analyzed comprised the ADI for anterior instability, C1/C2 alignment and lateral mass joint congruency for dislocation characterization, and vertebral artery morphology and anatomical variations to assess surgical risk. MRI was additionally employed to evaluate spinal cord compression and identify associated abnormalities, such as cerebellar tonsillar herniation or syringomyelia. Together, these comprehensive preoperative evaluations provided an objective foundation for diagnosis confirmation, surgical indication determination, and optimal fusion strategy planning for each patient.

### 2.2 Statistical Analysis

Statistical analyses were performed using IBM SPSS Statistics version 26.0 (IBM Corp., Armonk, NY, USA). Continuous variables were assessed for normality using the Shapiro–Wilk test. Normally distributed continuous variables are presented as mean ± standard deviation, whereas non‐normally distributed variables are presented as median and interquartile range when appropriate. Categorical variables are expressed as number and percentage. Paired continuous variables before and after surgery were compared using the paired‐samples *t*‐test or Wilcoxon signed‐rank test, as appropriate. Comparisons between the endoscopic‐assisted and open‐surgery groups were performed using the independent‐samples *t*‐test or Mann–Whitney *U* test for continuous variables and the chi‐square test or Fisher's exact test for categorical variables. For clinical outcomes measured at multiple postoperative time points, including JOA, VAS, and NDI scores, repeated‐measures analysis of variance was used when the assumptions of normality and sphericity were satisfied; otherwise, the Friedman test was applied. When appropriate, post hoc pairwise comparisons were performed with Bonferroni correction. A two‐sided *p*‐value < 0.05 was considered statistically significant.

### 2.3 Surgical Technique

#### 2.3.1 Patient Positioning and Anesthesia

Patients were placed in the prone position on a Jackson spinal table following induction of general anesthesia. The head was carefully secured in a Mayfield headrest to provide rigid fixation while ensuring adequate protection of the cranial vault and facial structures. Special attention was paid to avoid excessive pressure on the eyes and facial soft tissues by using soft protective padding, and the eyes were checked preoperatively and periodically intraoperatively to ensure they were free from compression and at neutral alignment.

**FIGURE 2:**
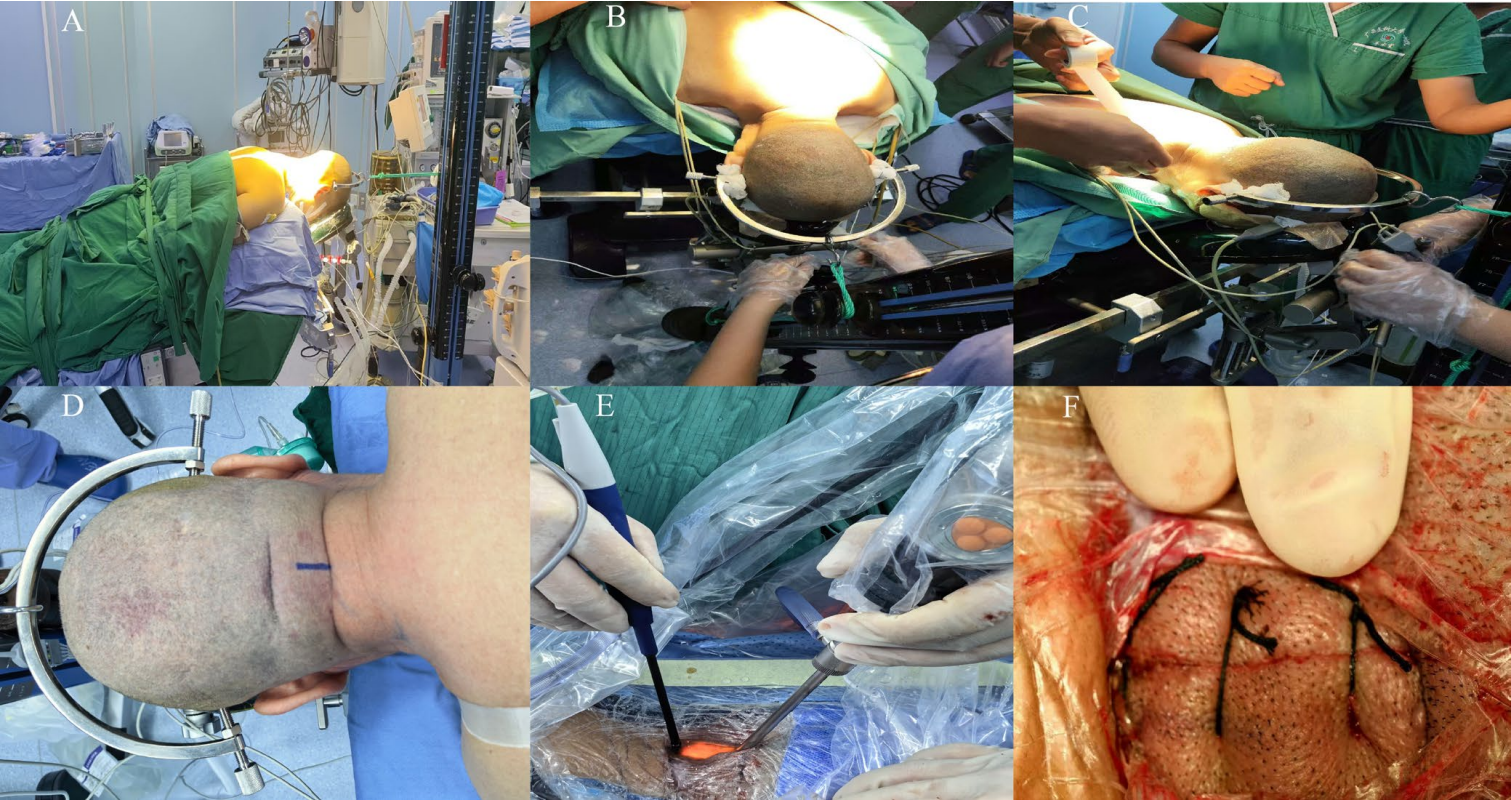
Patient positioning, cranial traction, and establishment of the minimally invasive endoscopic working corridor. (A) The patient was placed in the prone position on a Jackson spinal table, with the head secured using a Mayfield head holder and the table adjusted to a reverse Trendelenburg position. (B, C) Cranial traction was applied through sterile traction cords connected to the Mayfield apparatus, with the traction vector aligned as parallel as possible to the C1–C2 joint axis. (D) The posterior cervical midline skin incision was planned and marked under fluoroscopic guidance. (E) Establishment of the endoscopic working channel through a small posterior cervical incision after sequential soft‐tissue dilation. (F) Intraoperative view showing the limited posterior cervical incision and minimally invasive operative corridor used for endoscopic‐assisted atlantoaxial fusion.

The surgical table was adjusted to a reverse Trendelenburg position [14], with the head elevated relative to the feet, to accomplish multiple goals: (1) reduce the risk of venous congestion and pressure‐related complications, (2) allow the cervical spine to remain as parallel as possible to the traction vector, and (3) facilitate optimal intraoperative endoscopic access and instrument maneuverability in the upper cervical region (Figure 2A–C).

Cranial traction was applied and maintained throughout the procedure using sterile traction cords connected to the Mayfield apparatus. The weight was individualized based on the degree of atlantoaxial dislocation observed on preoperative imaging, with a maximum traction load not exceeding one‐sixth of the patient's body weight [15]. The traction vector was aligned in parallel with the C1–C2 joint axis to assist with joint reduction while minimizing cervical strain.

#### 2.3.2 Endoscopic Channel Establishment

Under C‐arm fluoroscopic guidance, the C1/C2 segment was accurately localized. Preoperatively, a longitudinal midline skin incision measuring 2.0–2.5 cm was planned and marked on the posterior cervical region (Figure 2D). Following general anesthesia and aseptic preparation, a skin incision was made along the marked line, and the superficial fascia was carefully incised. Subsequently, the subcutaneous soft tissues were dissected laterally by 1.5–2.0 cm to expose the paravertebral muscle space.

**FIGURE 3:**
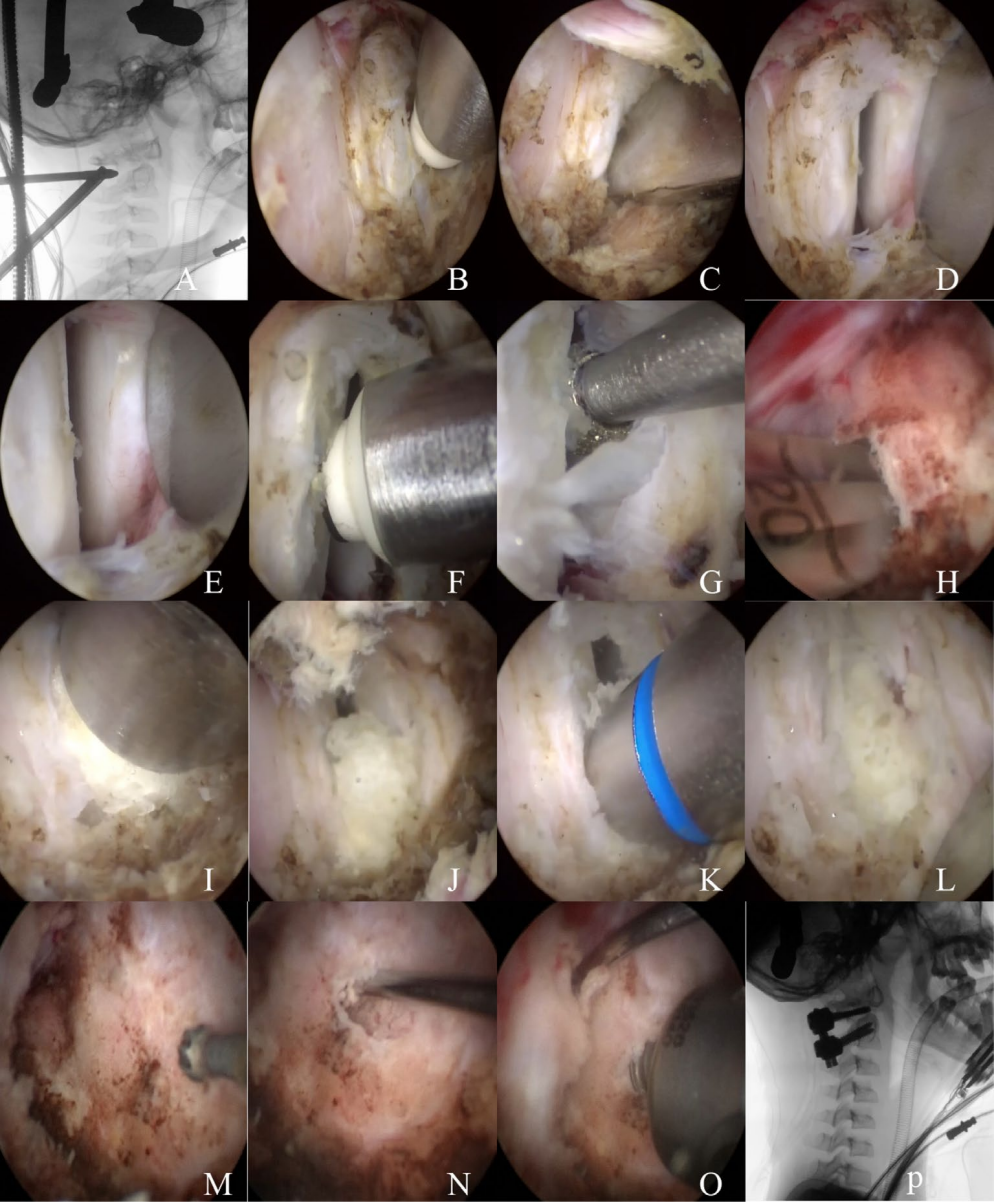
Step‐by‐step endoscopic‐assisted posterior atlantoaxial lateral mass fusion procedure. (A) Intraoperative C‐arm fluoroscopy was used to confirm localization of the C1–C2 segment and the trajectory of the working channel. (B) Endoscopic removal of soft tissue around the C2 isthmus. (C, D) Subperiosteal dissection and exposure of the bony boundaries of the C1–C2 lateral mass joint. (E) Identification and protection of the C2 nerve root and adjacent venous plexus under endoscopic visualization. (F, G) Excision of the joint capsule and decortication of the articular surface using endoscopic instruments and a high‐speed burr. (H) Gentle distraction and mobilization of the C1–C2 joint space to facilitate joint release and fusion bed preparation. (I, J) Delivery of allogeneic cancellous bone granules into the prepared atlantoaxial joint space through a cannula and pusher system. (K) Controlled compaction of the bone graft under direct endoscopic visualization. (L) Endoscopic confirmation of adequate graft filling within the atlantoaxial joint space. (M) Identification of the anatomical landmarks for C1 lateral mass and C2 pedicle screw entry points. (N) Preparation of the screw trajectory by drilling and tapping under endoscopic guidance. (O) Insertion of C1 lateral mass and C2 pedicle screws through the established minimally invasive corridor. (P) Final intraoperative fluoroscopic image confirming satisfactory screw–rod fixation and atlantoaxial alignment.

Blunt separation of the trapezius, splenius capitis, and semispinalis capitis muscles was performed along their natural fiber planes using a guiding rod. Sequential soft tissue dilators were then inserted to establish a minimally invasive working channel. Through this channel, the endoscopic system and operating instruments were introduced. Intraoperative photographs demonstrate the surgeon performing the procedure through the small 1.5–2.0 cm incision (Figure 2E,F). Throughout the procedure, real‐time C‐arm fluoroscopy (Figure 3A) was utilized to guide and confirm the precise positioning of the guiding rod, dilation channel, and endoscope, ensuring optimal trajectory and safety. For transparency, some intraoperative images in Figure 3 were selected from our earlier technical case report to illustrate key steps of the surgical procedure. The panels shown here were newly selected from the original operative record and are not direct duplicates of the previously published figure panels [13].

#### 2.3.3 C1/C2 Joint Exposure and Preparation

Continuous irrigation was maintained throughout the procedure to ensure a clear and stable endoscopic field. Soft tissues surrounding the C2 isthmus were carefully removed using electrocautery, as shown in Figure 3B. Subperiosteal dissection was then performed to expose the bony boundaries, providing essential anatomical landmarks for precise localization of the atlantoaxial joint capsule, as illustrated in Figure 3C,D. Under endoscopic visualization, the C2 nerve root and accompanying venous plexus were gently retracted with a nerve retractor to prevent neurovascular injury. The joint capsule was subsequently excised, and hypertrophic bone along the joint margins was debrided using a high‐speed burr to fully expose the subchondral bone, thereby maximizing joint space visualization and ensuring safe manipulation under continuous endoscopic guidance, as demonstrated in Figure 3E–G.

After adequate preparation of the articular surfaces, the joint space was gently distracted and mobilized using a nerve dissector or elevator to achieve sufficient joint release and ensure complete exposure of the lateral mass articular cavity, as shown in Figure 3H. This maneuver facilitated subsequent bone graft insertion and enhanced fusion bed preparation while minimizing the risk of excessive traction or neural irritation.

#### 2.3.4 Bone Grafting

Allogeneic cancellous bone granules measuring 2–5 mm were grafted into the prepared joint space through a delivery cannula and pusher system under continuous endoscopic visualization, as shown in Figure 3I,J. Throughout the procedure, meticulous care was taken to prevent graft spillage and to protect adjacent neurovascular structures. The bone graft volume and degree of compaction were continuously monitored in real time to ensure sufficient filling and stable contact between the articular surfaces without causing excessive joint distraction, as illustrated in Figure 3K. In most cases, unilateral fusion provided adequate stability; however, bilateral fusion was performed when additional biomechanical support was required (Figure 3L).

#### 2.3.5 Screw Placement

Utilizing the established endoscopic portal, the anatomical landmarks for the C1 lateral mass and C2 pedicle screw entry points were carefully identified as shown in Figure 3M. A high‐speed burr was employed to create pilot holes at the predetermined trajectories, ensuring accurate angulation and depth. Sequential tapping was then performed to prepare the screw pathways in a controlled manner, minimizing cortical breach and thermal injury as shown in Figure 3N. C1 lateral mass and C2 pedicle screws were subsequently inserted under continuous endoscopic monitoring, allowing real‐time confirmation of screw trajectory and preventing neurovascular compromise as shown in Figure 3O. After satisfactory screw placement, pre‐bent titanium rods were positioned and appropriately compressed to achieve rigid fixation and restore atlantoaxial alignment as shown in Figure 3P. Final inspection confirmed construct stability. Hemostasis was achieved, residual bone debris was meticulously removed, and a drainage tube was placed if necessary to prevent postoperative hematoma formation.

#### 2.3.6 Postoperative Imaging and Rehabilitation

**FIGURE 4:**
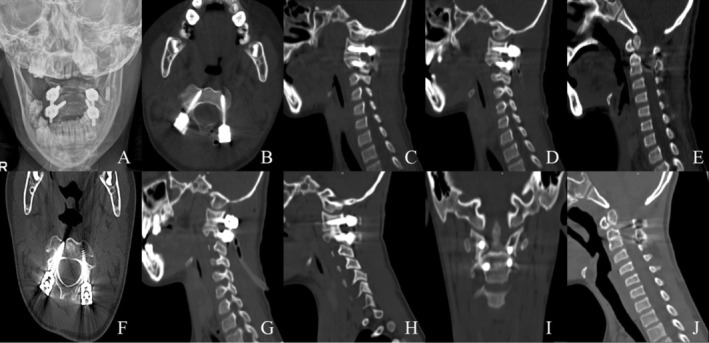
Postoperative radiographic and CT evaluation after endoscopic‐assisted posterior atlantoaxial fusion. (A) Postoperative open‐mouth odontoid radiograph showing bilateral C1–C2 screw–rod fixation and restoration of atlantoaxial alignment. (B) Axial CT image showing the position of the C1 lateral mass and C2 pedicle screws. (C, D) Sagittal CT reconstructions showing screw placement, reduction of atlantoaxial dislocation, and restoration of the C1–C2 relationship. (E) Sagittal CT image showing the craniovertebral junction after fixation and decompression. (F) Axial CT image demonstrating bilateral screw trajectories and the relationship between the implants and surrounding osseous structures. (G, H) Sagittal CT images showing the atlantoaxial joint space and intra‐articular graft placement. (I) Coronal CT reconstruction showing bilateral C1–C2 fixation and alignment. (J) Sagittal CT reconstruction showing maintained cervical alignment after posterior atlantoaxial fusion.

Postoperative radiographs and computed tomography (CT) scans were performed to evaluate the accuracy of screw placement, the condition of bone grafting, and the degree of joint distraction. As shown in Figure 4, postoperative imaging demonstrated satisfactory screw positioning, stable graft filling within the atlantoaxial joint space, and improved joint opening compared with preoperative findings, indicating effective decompression and successful fusion preparation. Patients subsequently received individualized rehabilitation programs emphasizing gradual cervical mobilization, pain management, and functional recovery under close clinical supervision.

## 3 Results

### 3.1 Demographics

A total of 16 patients (7 males and 9 females) underwent endoscopic‐assisted posterior atlantoaxial fusion, with a mean age of 32.38 ± 18.93 years (range, 6–55). Baseline characteristics, including height, weight, and disease duration, are summarized in Table 2. The mean height was 157.06 ± 19.09 cm, the mean weight was 53.81 ± 18.80 kg, and the mean disease duration was 9.69 ± 5.74 months.

**TABLE 2:** Descriptive statistics of demographics and surgical data.

Variable	Mean ± SD (range)
Age (years)	32.38 ± 18.93 (6–55)
Height (cm)	157.06 ± 19.09 (118–175)
Weight (kg)	53.81 ± 18.80 (22–72)
Disease duration (months)	9.69 ± 5.74 (3–24)
Intraoperative blood loss (mL)	40.06 ± 9.28 (25–60)
Operation time (min)	103.06 ± 9.55 (90–125)

The mean operative time was 103.06 ± 9.55 min, and the mean intraoperative blood loss was 40.06 ± 9.28 mL. All procedures were completed successfully without intraoperative neurovascular injury or hardware malposition. These data are summarized in Table 2.

### 3.2 Radiographic Parameters

Postoperative radiological assessment demonstrated significant correction of atlantoaxial alignment. The atlantodental interval (ADI) decreased from 5.14 ± 1.51 mm preoperatively to 3.37 ± 0.52 mm postoperatively (*p* < 0.001), indicating satisfactory reduction of the dislocation. The C1–C2 joint height increased from 2.98 ± 0.27 mm to 4.37 ± 0.49 mm (*p* < 0.001), reflecting effective restoration of the joint space following intra‐articular bone grafting and fixation. Follow‐up CT confirmed bony bridging at the fusion site in nearly all patients. Detailed radiographic and alignment parameters are summarized in Table 3.

**TABLE 3:** Radiographic parameters pre‐ and postoperatively.

Parameter	Preoperative (mean ± SD)	Postoperative (mean ± SD)	*p*
ADI (mm)	5.14 ± 1.51	3.37 ± 0.52	**< 0.001**
C1–C2 joint height (mm)	2.98 ± 0.27	4.37 ± 0.49	**< 0.001**

### 3.3 Postoperative Outcomes and Complications

Clinical outcomes improved after surgery and remained better than preoperative values during follow‐up. As shown in Table 4, the mean JOA score increased from 8.44 ± 0.89 preoperatively to 13.44 ± 0.89 immediately postoperatively, 14.44 ± 0.89 at 1 month, and 13.31 ± 1.20 at 3 months. The mean VAS score decreased from 8.00 ± 0.73 preoperatively to 4.12 ± 0.62 immediately postoperatively, 3.25 ± 0.45 at 1 month, and 2.25 ± 0.45 at 3 months. The mean NDI score decreased from 31.88 ± 1.93 preoperatively to 20.06 ± 1.24 immediately postoperatively, 15.31 ± 0.95 at 1 month, and 12.88 ± 1.26 at 3 months. These findings indicate postoperative neurological improvement, pain relief, and functional recovery.

**TABLE 4:** Functional outcomes (JOA, VAS, NDI).

Parameter	Pre‐op	Post‐op	1 month	3 months	*p*
JOA score	8.44 ± 0.89	13.44 ± 0.89	14.44 ± 0.89	13.31 ± 1.20	**< 0.001**
VAS	8.00 ± 0.73	4.12 ± 0.62	3.25 ± 0.45	2.25 ± 0.45	**< 0.001**
NDI	31.88 ± 1.93	20.06 ± 1.24	15.31 ± 0.95	12.88 ± 1.26	**< 0.001**

Bone fusion (Table 5) was achieved in 15 patients (93.8%) at 3 months, while one patient (6.2%) demonstrated partial fusion but achieved complete union by 6 months. Minor postoperative complications included mild pain in 4 patients (25%) and small local hematoma in 1 patient (6.2%), all of which resolved with conservative management. No cases of cerebrospinal fluid leakage, infection, or screw loosening were observed. By the final follow‐up, all 16 patients (100%) exhibited improvement in both sensory and motor function. Representative imaging demonstrated satisfactory reduction, stable fixation, and continuous osseous fusion at the C1–C2 lateral mass joint.

**TABLE 5:** Postoperative outcomes and complications.

Parameter	Value/*n* (%)
Bone fusion at 3 months	Fused 15 (93.8%), Partial 1 (6.2%)
Postoperative complications	Mild pain 4 (25%), Mild hematoma 1 (6.2%), None 11 (68.8%)
Sensory disturbance (at follow‐up)	16 (100%) improved
Motor dysfunction (at follow‐up)	16 (100%) improved

### 3.4 Comparison Between Endoscopic‐Assisted and Open Posterior Fusion Outcomes

To further evaluate the clinical utility of the endoscopic‐assisted technique, a control group of 20 patients who underwent conventional open posterior atlantoaxial fusion was included. Demographic and clinical characteristics of the open group are detailed in Table S1, and a comparative summary between both groups is presented in Table 6.

**TABLE 6:** Comparison between endoscopic‐assisted and open posterior atlantoaxial fusion.

Variable	Endoscopic (mean ± SD)	Open (mean ± SD)	*p*
Age (years)	32.38 ± 18.93	36.00 ± 17.84	0.562
Height (cm)	157.06 ± 19.09	158.95 ± 16.95	0.759
Weight (kg)	53.81 ± 18.80	56.20 ± 17.61	0.700
Pre‐op ADI (mm)	5.14 ± 1.51	5.39 ± 1.59	0.631
Post‐op ADI (mm)	3.37 ± 0.52	3.40 ± 0.53	0.846
Pre‐op C1‐C2 joint height (mm)	2.98 ± 0.27	3.04 ± 0.25	0.509
Post‐op C1‐C2 joint height (mm)	4.37 ± 0.49	4.49 ± 0.50	0.473
Disease duration (m)	9.69 ± 5.74	12.00 ± 6.56	0.268
Blood loss (mL)	40.06 ± 9.28	172.85 ± 13.13	< 0.01
Operation time (min)	103.06 ± 9.55	89.90 ± 3.26	< 0.01
JOA_Pre	8.44 ± 0.89	8.30 ± 1.13	0.686
JOA_Post	13.44 ± 0.89	11.30 ± 1.13	< 0.01
JOA_1M	14.44 ± 0.89	12.30 ± 1.13	< 0.01
JOA_3M	13.31 ± 1.20	14.30 ± 1.13	0.017
VAS_Pre	8.00 ± 0.73	7.90 ± 0.55	0.654
VAS_Post	4.12 ± 0.62	5.90 ± 0.55	< 0.01
VAS_1M	3.25 ± 0.45	4.90 ± 0.55	< 0.01
VAS_3M	2.25 ± 0.45	2.90 ± 0.55	< 0.01
NDI_Pre	31.88 ± 1.93	32.90 ± 1.83	0.115
NDI_Post	20.06 ± 1.24	20.55 ± 1.39	0.275
NDI_1M	15.31 ± 0.95	15.75 ± 1.07	0.202
NDI_3M	12.88 ± 1.26	13.35 ± 1.50	0.308

No statistically significant differences were found between the two groups in baseline variables, including age, height, weight, preoperative atlantodental interval (ADI), C1–C2 joint height, or disease duration (*p* > 0.05 for all), suggesting no statistically detectable baseline differences between the two groups.

Intraoperative outcomes revealed that the endoscopic group experienced significantly reduced blood loss (40.06 ± 9.28 mL vs. 172.85 ± 13.13 mL, *p* < 0.01), consistent with the minimally invasive nature of the technique. However, the mean operative time was slightly longer than that of the open group (103.06 ± 9.55 min vs. 89.90 ± 3.26 min, *p* < 0.01).

**FIGURE 5:**
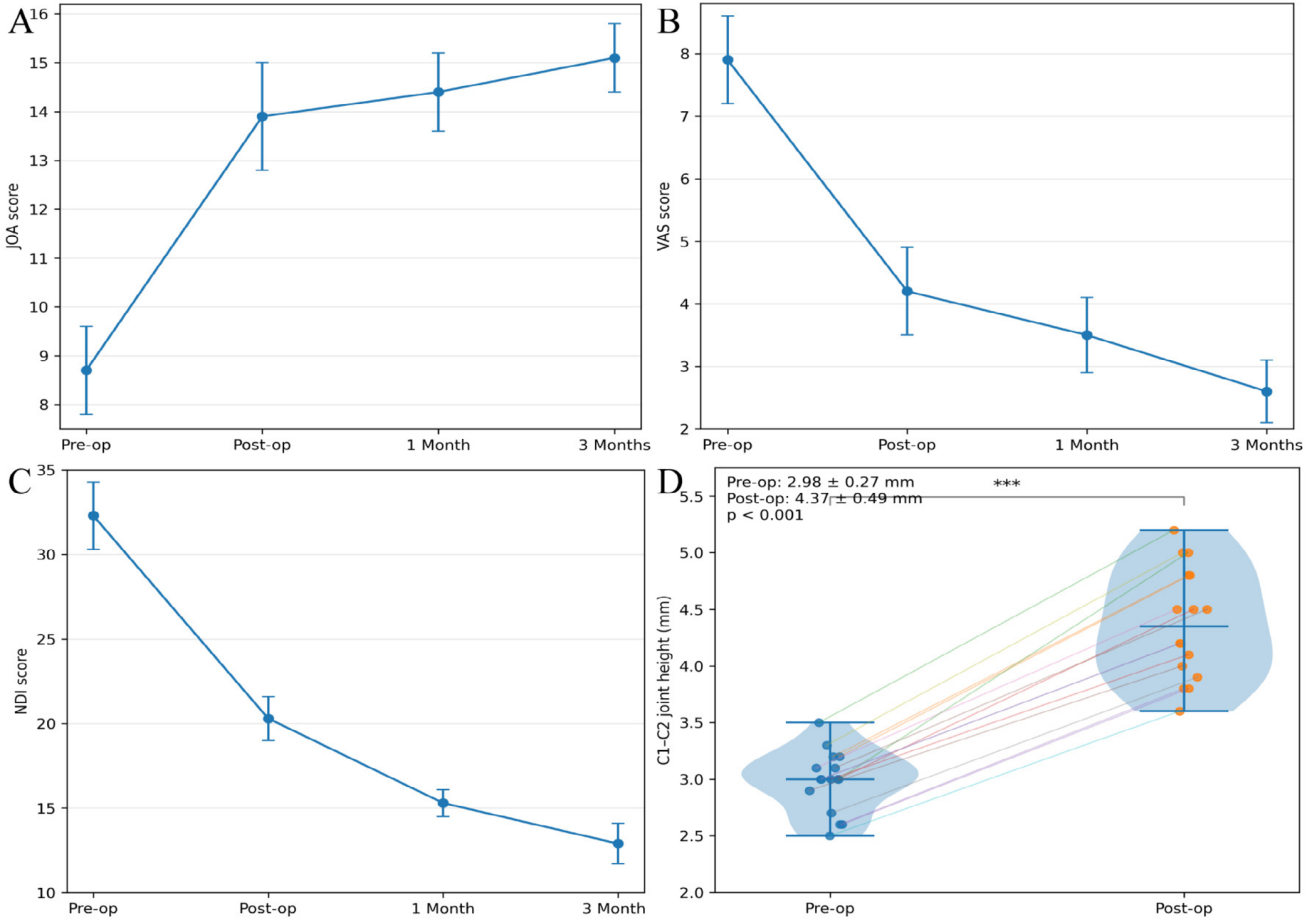
Changes in clinical outcomes and C1–C2 joint height after endoscopic‐assisted posterior atlantoaxial fusion. (A) Japanese Orthopedic Association (JOA) scores improved after surgery and remained higher than preoperative values during follow‐up. (B) Visual analog scale (VAS) scores decreased after surgery, reflecting progressive postoperative pain relief during follow‐up. (C) Neck Disability Index (NDI) scores decreased after surgery, indicating improvement in neck‐related functional disability. (D) Violin plot showing the distribution of C1–C2 joint height before and after surgery based on individual patient data; paired lines indicate within‐patient changes. Data in panels A–C are presented as mean ± standard deviation. JOA, Japanese Orthopedic Association; NDI, neck disability index; VAS, visual analog scale.

Regarding neurological function, the endoscopic group demonstrated superior early improvements in JOA scores at immediate postoperative and 1‐month follow‐up assessments (*p* < 0.01). Interestingly, the open group showed a modest advantage in JOA score at 3 months (14.30 ± 1.13 vs. 13.31 ± 1.20, *p* = 0.017), suggesting that neurological recovery patterns may differ between the two approaches and should be interpreted cautiously. Pain outcomes assessed via VAS were consistently better in the endoscopic group across all postoperative timepoints (*p* < 0.01). NDI scores showed no significant intergroup differences throughout follow‐up, indicating comparable functional neck recovery by 3 months postoperatively (Figure 5).

### 3.5 Illustrative Cases

#### 3.5.1 Case 1

A female patient with a type II odontoid (dens) fracture presented with atlantoaxial instability and mild neurological deficits. Preoperative imaging revealed anterior displacement of C1 over C2 with an atlantodental interval (ADI) of 4.8 mm and a reduced C1–C2 joint height of 3.0 mm. MRI showed no severe spinal cord compression, but mild motor dysfunction and sensory disturbances were noted. The patient underwent endoscopic‐assisted posterior atlantoaxial lateral mass fusion.

**FIGURE 6:**
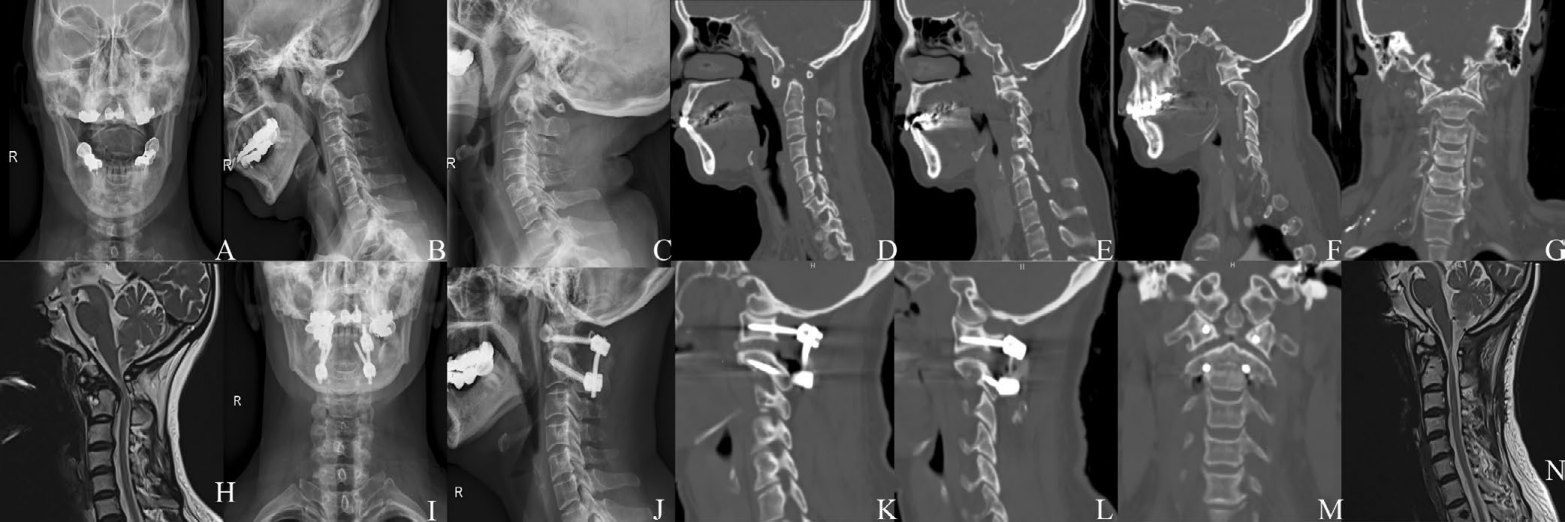
Illustrative Case 1: Type II odontoid fracture with atlantoaxial instability treated by endoscopic‐assisted posterior atlantoaxial fusion. (A) Preoperative open‐mouth odontoid radiograph showing atlantoaxial malalignment. (B, C) Preoperative dynamic lateral radiographs showing atlantoaxial instability and reducibility. (D, E) Preoperative sagittal CT reconstructions showing the odontoid fracture and abnormal C1–C2 alignment. (F) Postoperative sagittal CT image showing reduction of the atlantoaxial dislocation and restoration of the C1–C2 relationship. (G) Coronal CT reconstruction showing the odontoid process and bilateral atlantoaxial joint alignment after reduction. (H) Preoperative sagittal MRI showing the spinal cord and craniovertebral junction. (I, J) Postoperative anteroposterior and lateral radiographs showing bilateral C1 lateral mass and C2 pedicle screw–rod fixation. (K, L) Postoperative sagittal CT reconstructions showing screw placement, joint space restoration, and intra‐articular bone grafting. (M) Coronal CT reconstruction showing bilateral fixation and graft placement across the C1–C2 lateral mass joint region. (N) Follow‐up sagittal MRI showing adequate decompression and maintained craniovertebral alignment.

Postoperative imaging (Figure 6) demonstrated precise placement of C1 lateral mass and C2 pedicle screws under direct endoscopic visualization, restoration of the C1–C2 joint height to 5.0 mm, and well‐compacted allogeneic bone graft within the joint space. Three‐month follow‐up CT confirmed continuous osseous bridging at the fusion site with maintained screw alignment and stable fixation. Clinically, the patient showed notable improvement in motor function and sensory recovery. JOA score improved from 7 preoperatively to 12 at 3 months, VAS decreased from 8 to 2, and NDI improved from 35 to 15. A mild postoperative hematoma was observed but resolved with conservative management.

#### 3.5.2 Case 2

**FIGURE 7:**
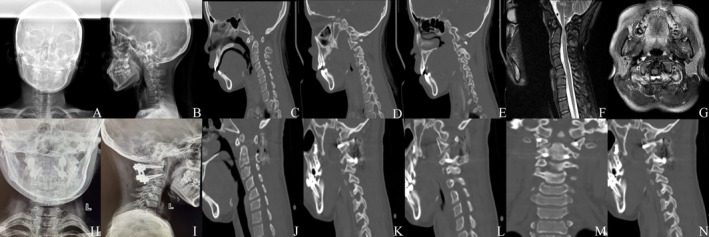
Illustrative Case 2: Type II odontoid fracture with atlantoaxial dislocation treated by endoscopic‐assisted posterior atlantoaxial fusion. (A) Preoperative anteroposterior cervical radiograph. (B) Preoperative lateral cervical radiograph. (C–E) Preoperative sagittal CT images demonstrating type II odontoid fracture and atlantoaxial dislocation. (F) Preoperative sagittal MRI showing the craniovertebral junction and spinal cord status. (G) Preoperative axial MRI showing the relationship between the odontoid process and the spinal cord. (H) Postoperative open‐mouth odontoid radiograph demonstrating satisfactory reduction and internal fixation. (I) Postoperative lateral cervical radiograph showing restoration of atlantoaxial alignment. (J–L) Postoperative sagittal CT images showing satisfactory reduction of the odontoid process, enlargement of the C1–C2 joint space, and good bone graft placement within the C1–C2 joint space. (M) Postoperative axial CT image confirming satisfactory reduction of the odontoid process. (N) Postoperative sagittal CT image showing good fusion of the C1–C2 grafted region.

A female patient with a type II odontoid fracture presented with atlantoaxial instability and incomplete neurological deficits. Preoperative radiographs demonstrated atlantoaxial malalignment on both anteroposterior and lateral views (Figure 7A,B). Preoperative sagittal CT images further confirmed type II odontoid fracture with C1–C2 dislocation and abnormal atlantoaxial alignment (Figure 7C–E). Preoperative MRI showed the craniovertebral junction and the relationship between the odontoid process and the spinal cord in both sagittal and axial views (Figure 7F,G). The patient underwent endoscopic‐assisted posterior atlantoaxial lateral mass fusion.

Postoperative open‐mouth and lateral radiographs demonstrated satisfactory reduction and stable C1 lateral mass–C2 pedicle screw fixation (Figure 7H,I). Postoperative sagittal CT images showed good bone graft placement within the C1–C2 joint space, enlargement of the atlantoaxial joint space, and satisfactory reduction of the odontoid process (Figure 7J–L). Axial CT confirmed satisfactory reduction of the odontoid process (Figure 7M), and sagittal CT showed good fusion of the grafted C1–C2 region (Figure 7N). Clinically, the patient experienced marked relief of neck pain and significant improvement in neurological function. The JOA score improved from 10 preoperatively to 15 at 3 months; the VAS score decreased from 8 to 2, and the NDI improved from 35 to 14. No postoperative complications were observed.

## 4 Discussion

### 4.1 Main Findings of This Study

This study introduced an endoscopic‐assisted posterior atlantoaxial lateral mass fusion technique for the treatment of atlantoaxial instability or dislocation and evaluated its early clinical and radiographic outcomes. The principal findings were as follows. First, all 16 procedures were completed successfully without intraoperative neurovascular injury or screw malposition, and no cerebrospinal fluid leakage, infection, or screw loosening was observed during follow‐up. Second, postoperative radiographic parameters showed significant improvement, with reduction of the atlantodental interval and restoration of C1–C2 joint height. Third, clinical outcomes, including JOA, VAS, and NDI scores, improved markedly during the early postoperative follow‐up period. Fourth, bony fusion was achieved in 93.8% of patients at 3 months, and the remaining patient achieved complete union by 6 months. Finally, compared with conventional open posterior atlantoaxial fusion, the endoscopic‐assisted technique was associated with significantly reduced intraoperative blood loss and better early postoperative pain relief, although it required a longer operative time. These findings suggest that endoscopic‐assisted posterior atlantoaxial fusion may provide a feasible minimally invasive option for selected patients with AAI/D, while maintaining the fundamental goals of posterior C1–C2 stabilization and fusion [16, 17, 18, 19].

### 4.2 Technical Rationale and Minimally Invasive Advantages

The technical rationale of this procedure is based on direct endoscopic visualization of the C1–C2 lateral mass joint, controlled preparation of the articular surface, and accurate intra‐articular bone grafting through a minimally invasive working corridor. Posterior atlantoaxial fusion requires a detailed understanding of the C1 posterior arch, C2 isthmus, C2 nerve root, venous plexus, lateral mass joint, and vertebral artery anatomy because anatomical variation in the craniovertebral junction may substantially increase surgical risk [16]. In conventional posterior atlantoaxial fusion, adequate exposure of these structures often requires extensive dissection of the posterior cervical musculature. Such exposure may contribute to intraoperative bleeding, postoperative axial pain, and soft‐tissue morbidity [17, 18].

In contrast, the endoscopic‐assisted approach allows the surgeon to reach the atlantoaxial joint through a limited incision and muscle‐splitting corridor. High‐definition endoscopic visualization facilitates identification of critical anatomical structures, including the C2 nerve root, venous plexus, joint capsule, and bony margins of the C1–C2 joint. This is consistent with the broader evolution of spinal endoscopic surgery, in which improved optics, working channels, and endoscopic instruments have expanded the application of endoscopic techniques from lumbar decompression to more complex cervical procedures [12, 20]. Continuous irrigation helps maintain a clear operative field and may improve hemostatic control. Moreover, direct visualization of joint surface preparation and bone graft delivery allows more controlled decortication and graft compaction, which may contribute to a favorable local fusion environment while reducing unnecessary soft‐tissue disruption [18, 19]. From a biological perspective, successful graft incorporation and bone fusion depend not only on mechanical stability but also on local osteogenic activity and vascularization, which are essential biological factors for bone regeneration and fusion maturation [21, 22].

### 4.3 Comparison With Conventional Open Posterior Fusion

The preliminary comparison with the open posterior fusion group provides clinically relevant information regarding the potential advantages and limitations of this endoscopic‐assisted technique. The most apparent advantage was the marked reduction in intraoperative blood loss in the endoscopic group, which is consistent with the minimally invasive nature of the procedure. Previous studies on minimally invasive atlantoaxial fusion and minimally invasive modification of the Goel–Harms technique have also emphasized that limited soft‐tissue dissection may reduce surgical exposure‐related bleeding and posterior cervical muscle injury [17, 18]. Therefore, our findings support the concept that targeted endoscopic exposure may decrease operative trauma while preserving the essential biomechanical principle of posterior C1–C2 fixation and fusion.

However, the operative time was longer in the endoscopic group than in the open group. This result is understandable because endoscopic‐assisted high cervical fusion requires careful channel establishment, repeated fluoroscopic confirmation, endoscopic identification of anatomical landmarks, controlled joint preparation, and technically demanding screw placement through a restricted corridor. Therefore, the longer operative time should not be interpreted as a failure of the technique, but rather as a reflection of its technical complexity and early learning curve.

In terms of clinical outcomes, the endoscopic group showed favorable early pain relief, as reflected by better postoperative VAS scores. This may be related to reduced muscle stripping and less posterior soft‐tissue injury. JOA scores showed early improvement in the endoscopic group, whereas the open group showed a modest advantage at 3 months. This finding indicates that the neurological recovery pattern may not be uniformly superior with the endoscopic technique and should be interpreted cautiously. NDI scores were comparable between the two groups during follow‐up, suggesting that both procedures can achieve functional improvement when appropriate stabilization and fusion are obtained. Overall, the current comparison suggests that endoscopic‐assisted posterior atlantoaxial fusion may offer advantages in reducing surgical trauma and early postoperative pain, but it should not yet be considered superior to open fusion in all clinical dimensions [18, 19].

### 4.4 Technical Challenges and Learning Curve

Despite its potential advantages, endoscopic‐assisted posterior atlantoaxial fusion is technically demanding. The C1–C2 region has a complex anatomical configuration, with a narrow working space and close proximity to the vertebral artery, C2 nerve root, venous plexus, spinal cord, and dural sac. In addition, accurate placement of C1 lateral mass screws and C2 pedicle screws requires precise anatomical orientation and repeated confirmation of screw trajectory. These anatomical and technical challenges are particularly relevant in the upper cervical spine, where vertebral artery variation and limited osseous corridors may increase the risk of neurovascular injury [16].

The restricted endoscopic corridor may also increase the difficulty of instrument manipulation, especially during joint capsule excision, articular surface decortication, bone graft delivery, and rod placement. Previous studies on posterior cervical endoscopic procedures have shown that endoscopic techniques are associated with a measurable learning curve, and stable procedural efficiency may require accumulation of operative experience [23]. Therefore, surgeons adopting this technique should have sufficient experience in both posterior atlantoaxial fixation and spinal endoscopic procedures. During the early stage of clinical application, careful patient selection is essential. Patients with reducible atlantoaxial dislocation, relatively preserved anatomical landmarks, and no severe congenital deformity may be more suitable candidates. In contrast, patients with severe basilar invagination, complex vertebral artery anomalies, comminuted fractures, or marked craniocervical deformity may require conventional open exposure, navigation‐assisted fixation, or individualized reconstructive strategies [16, 24, 25].

### 4.5 Limitations and Future Perspectives

Several limitations should be acknowledged. First, the sample size was relatively small, and the follow‐up duration was limited; therefore, the long‐term fusion rate, implant stability, and functional durability require further evaluation. Second, although a conventional open posterior fusion group was included for preliminary comparison, the study was retrospective and non‐randomized, and the two groups were not strictly matched. Potential selection bias and residual confounding therefore cannot be excluded. Third, all procedures were performed by experienced surgeons at a single center, which may limit the generalizability of the results. Fourth, biomechanical testing was not performed, and the stability of this construct under different loading conditions remains to be further investigated.

Future studies should include larger multicenter cohorts, longer follow‐up, and more rigorous comparative designs. Propensity score matching or prospective controlled studies may help clarify the relative benefits of endoscopic‐assisted and open posterior atlantoaxial fusion. In addition, integration of navigation, robotic assistance, three‐dimensional planning, and augmented reality may further improve the accuracy and safety of this technique, particularly in patients with complex anatomy or high‐risk vertebral artery variations [24, 25]. Radiation exposure related to repeated fluoroscopic confirmation is another important consideration, and navigation‐assisted fluoroscopy may help reduce radiation exposure during minimally invasive spine surgery [26].

## 5 Conclusion

Endoscopic‐assisted posterior atlantoaxial lateral mass fusion represents a safe and feasible minimally invasive technique for the management of atlantoaxial instability or dislocation. This approach enables accurate screw placement, effective restoration of C1–C2 alignment, and reliable bone fusion while minimizing soft‐tissue trauma, intraoperative blood loss, and postoperative pain. Early clinical outcomes demonstrate rapid neurological and functional recovery, highlighting its potential as an alternative to conventional open posterior fusion. Despite technical challenges and a limited working corridor, careful patient selection and meticulous endoscopic technique can achieve satisfactory results. Further studies with larger cohorts, longer follow‐up, and comparative designs are warranted to confirm long‐term efficacy, optimize surgical protocols, and expand the clinical applicability of this approach.

## Author Contributions

**Dequan Liu:** data curation. **Chengqian Huang:** methodology. **Jiang Xue:** conceptualization. **Jichong Zhu:** writing – original draft, visualization, supervision. **Jiarui Chen:** software. **Chong Liu:** writing – review and editing. **Xinli Zhan:** writing – review and editing. **Yufan Xu:** formal analysis.

## Funding

This study was supported by the National Natural Science Foundation of China (Grant No. NSFC82360422) and the Joint Project on Regional High‐Incidence Diseases Research of the Guangxi Natural Science Foundation (Grant No. 2024GXNSFAA010073).

## Ethics Statement

This study was reviewed and approved by the Ethics Committee (2025‐E0882). All procedures involving human participants were conducted in accordance with the ethical standards of the institutional and national research committees and with the 1964 Helsinki Declaration and its later amendments. Written informed consent was obtained from all participants or their legal guardians prior to enrollment. Patient identities and personal information were anonymized and kept strictly confidential throughout the study to ensure privacy protection.

## Conflicts of Interest

The authors declare no conflicts of interest.

## Supporting information


**Table S1:** Open surgery cases.

## Data Availability

The datasets generated and analyzed during the current study are available from the corresponding author on reasonable request. All relevant data supporting the findings of this study, including anonymized patient records and imaging datasets, have been securely archived at the First Affiliated Hospital of Guangxi Medical University and can be accessed upon institutional approval.
